# Supplementary material for: A wheat resistosome defines common principles of immune receptor channels
Source: Nature. 2022 Sep 26;610(7932):532–9. doi: 10.1038/s41586-022-05231-w (PMC9581773; doi:10.1038/s41586-022-05231-w)
Supplement: Supplementary file 1 — Supplementary Figs.1–11. [file 41586_2022_5231_MOESM1_ESM.pdf]

nature portfolio

https://doi.org/10.1038/s41586-022-05231-w

Supplementary information

A wheat resistosome defines common principles of immune receptor channels

In the format provided by the
authors and unedited

oligomerization mutants

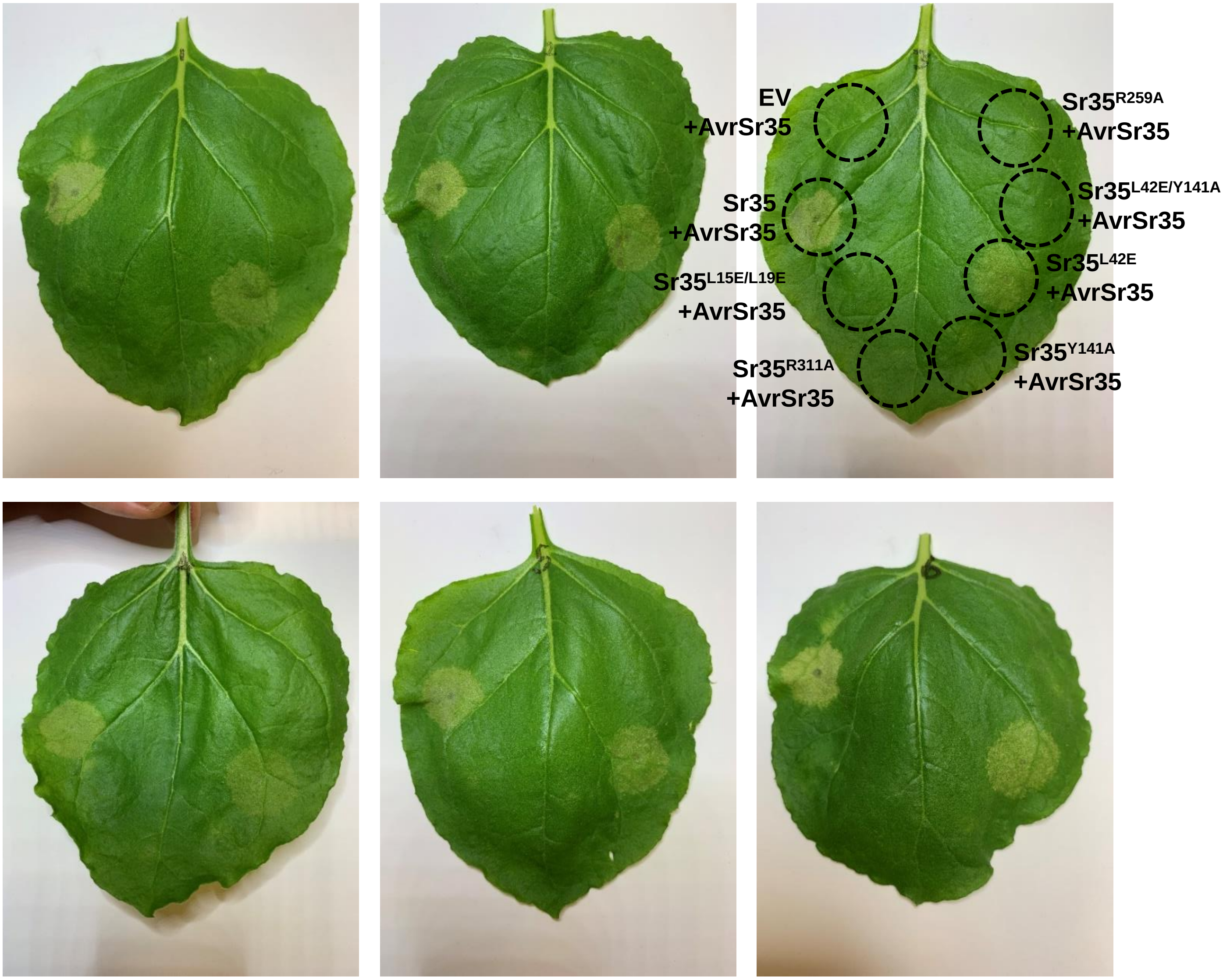

Supplemental Information 1 Tobacco cell death data of inter-protomer CC domain and NBD mutants corresponding to Figure 2h

EDVID mutants

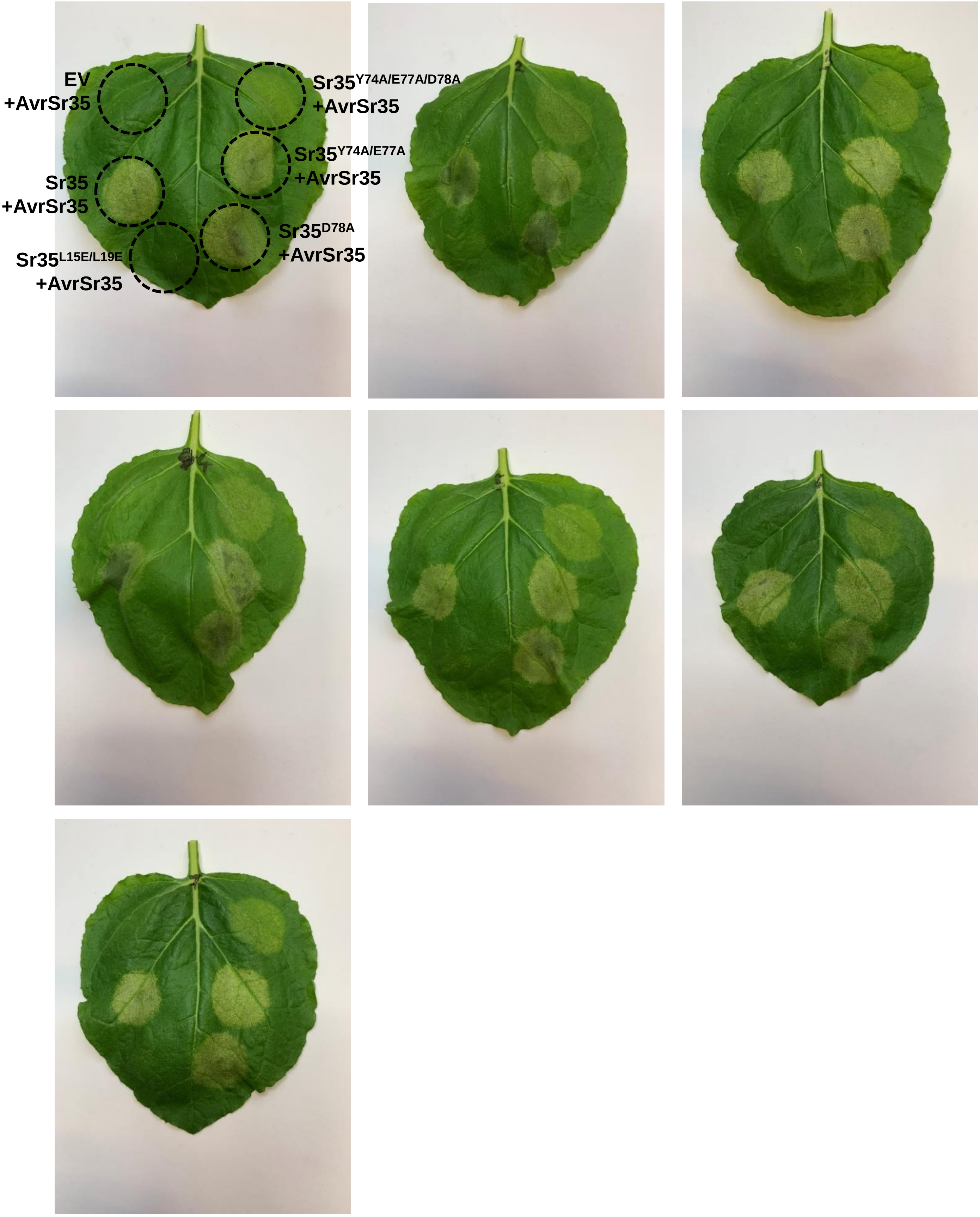

R-cluster mutants

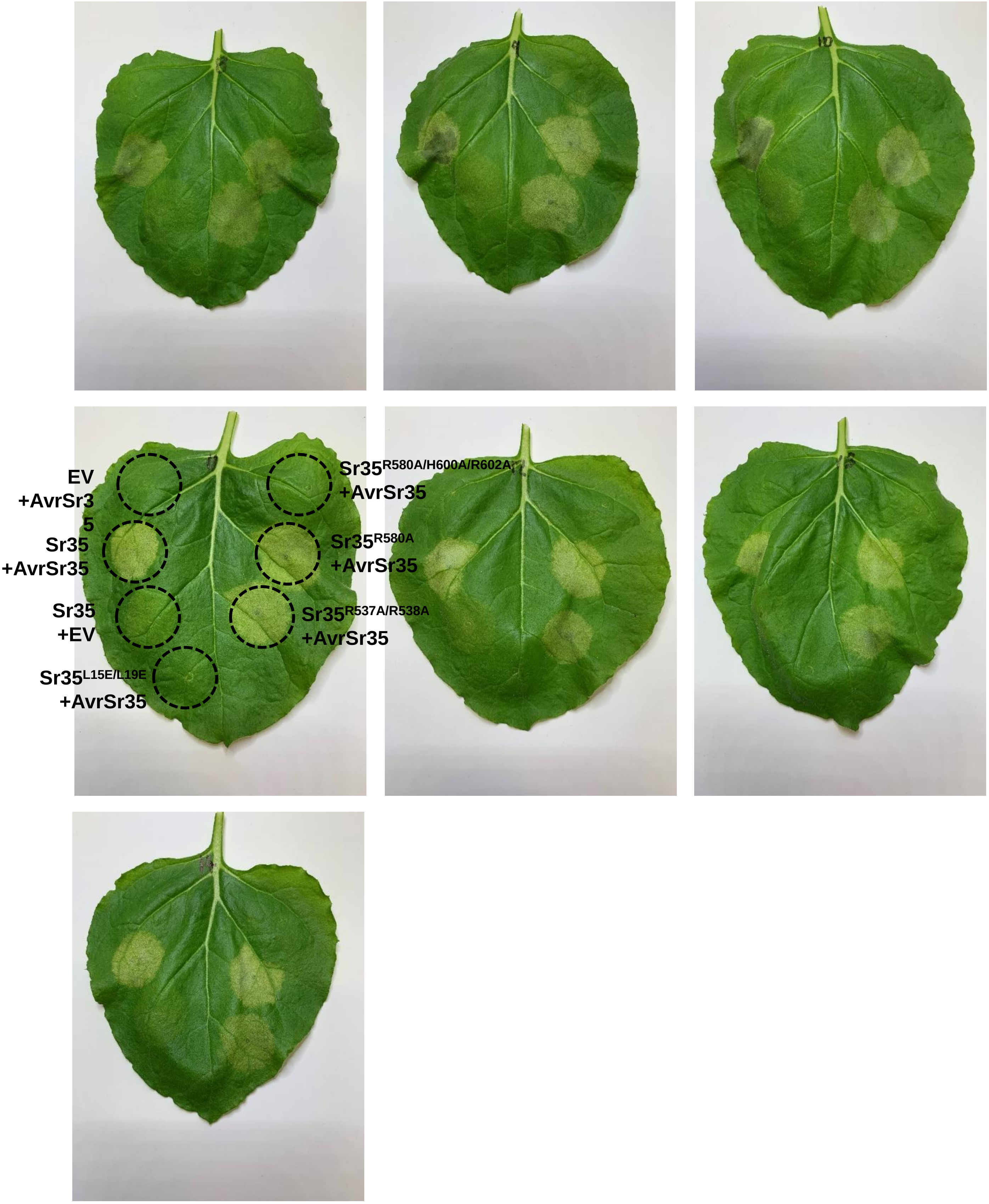

Supplemental Information 2 Tobacco cell death data of EDVID motif and R-cluster corresponding to Figure 2j

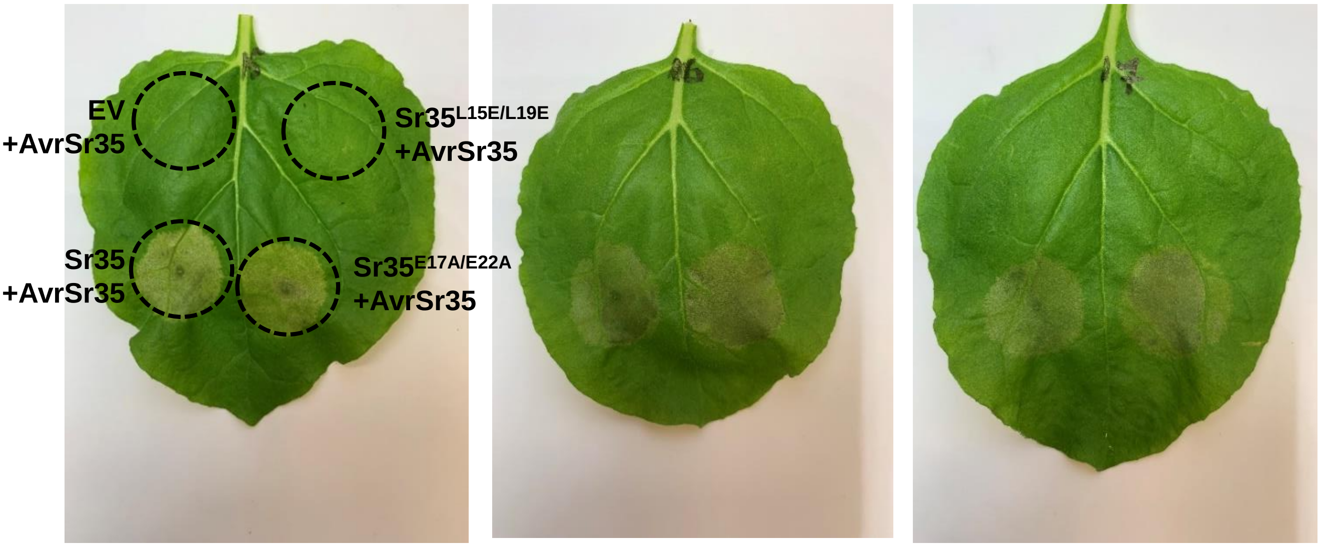

Supplemental Information 3 Tobacco cell death data of Sr35 channel mutants corresponding to Figure 3e

Sr35 LRR mutants

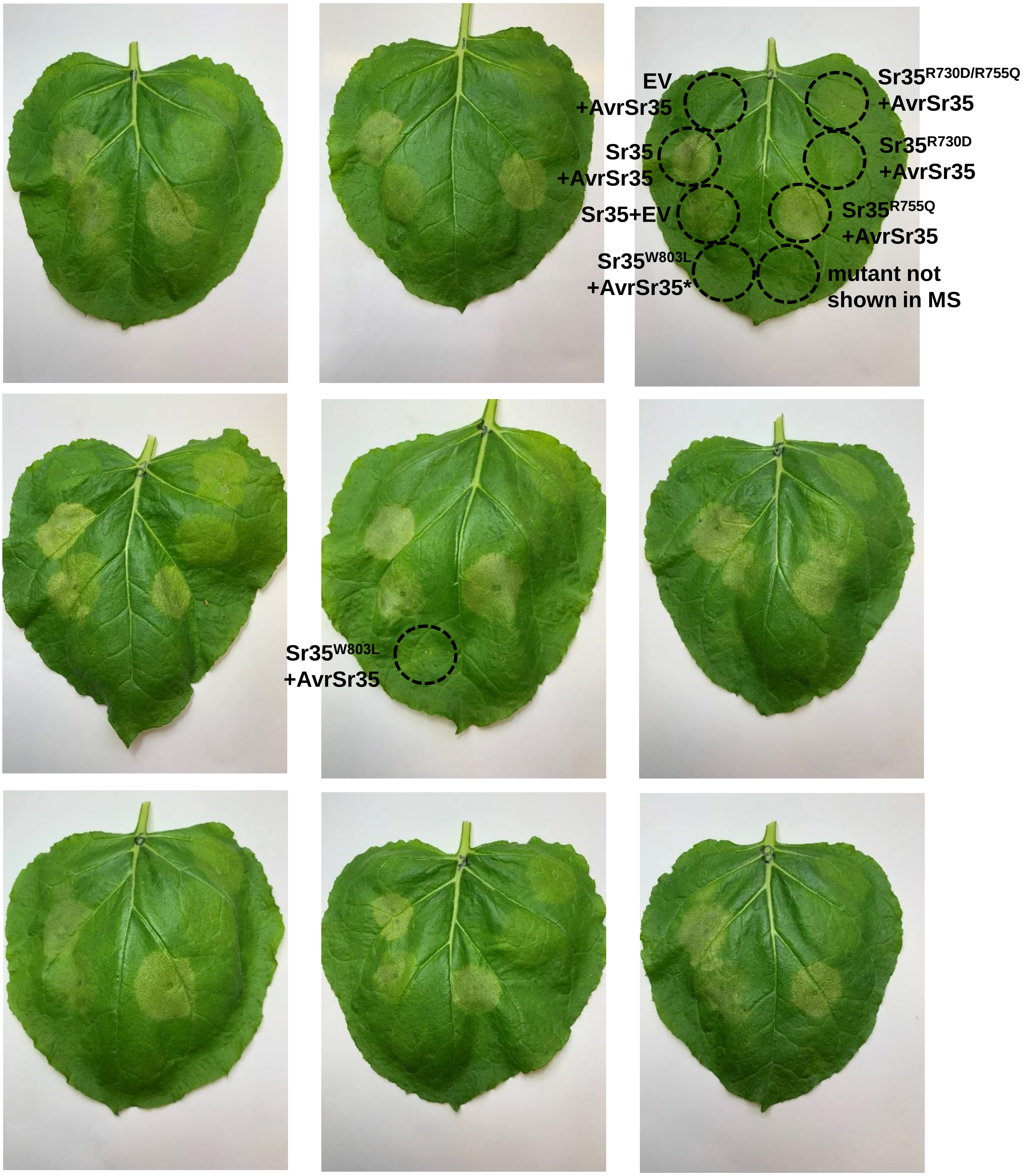

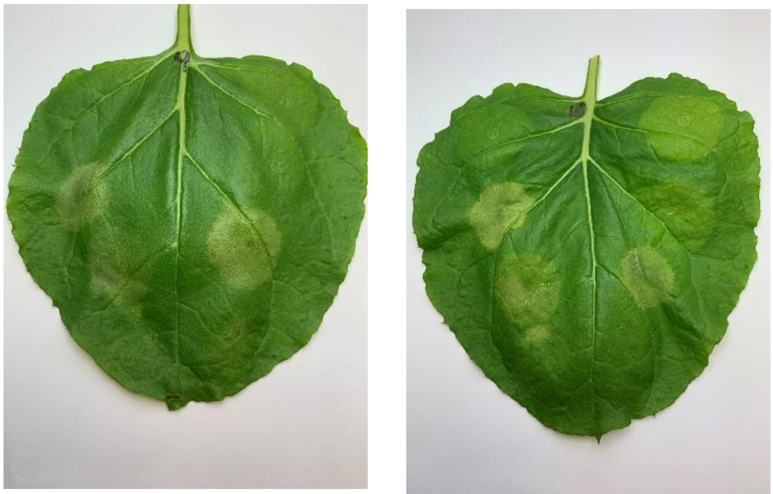

*shown in MS
from leave #4

Supplemental Information 4
Tobacco cell death data of Sr35 LRR mutants corresponding to Figure 4d. *shown in article from leave #4

AvrSr35 mutants

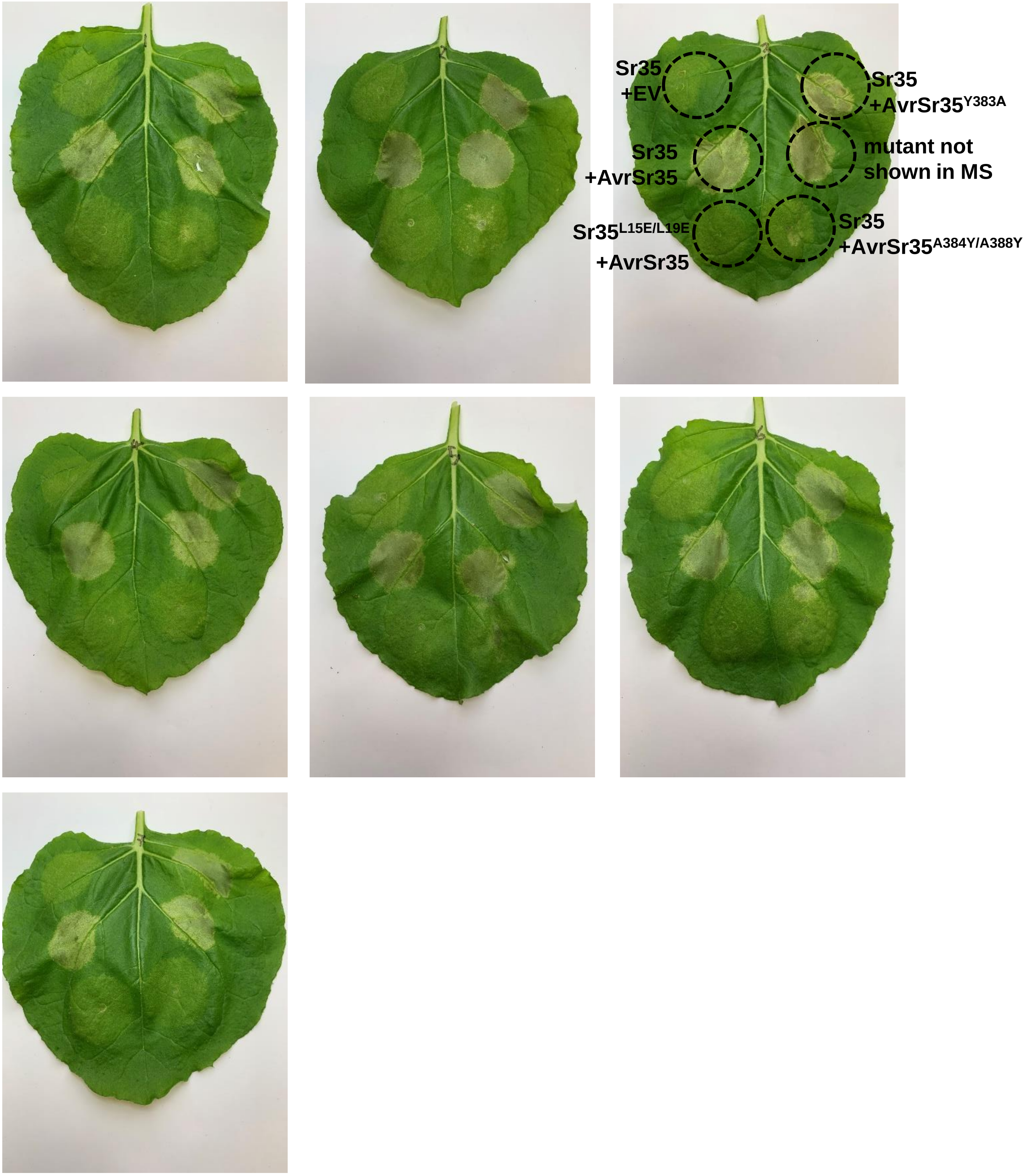

AvrSr35 mutants

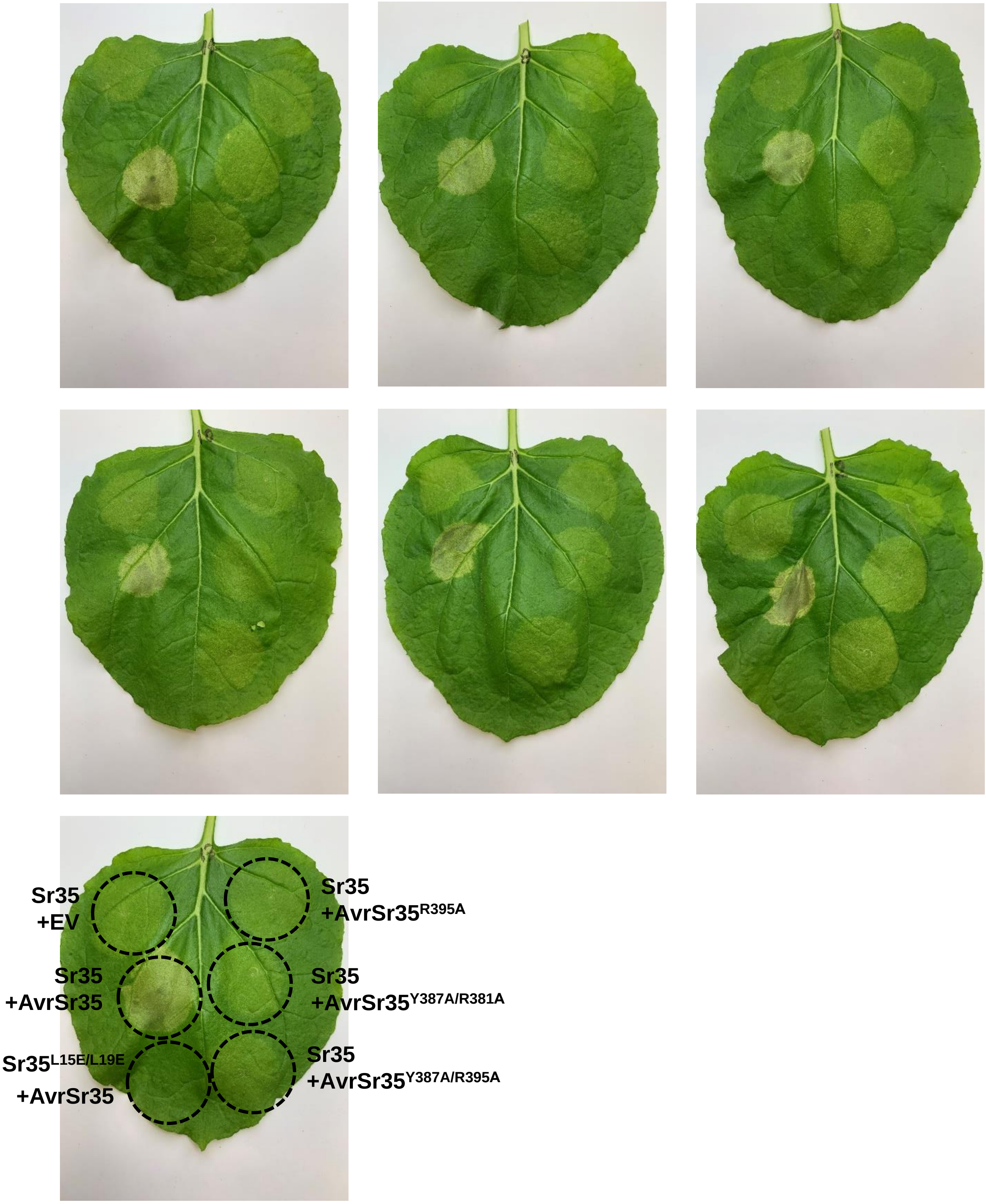

AvrSr35 mutants

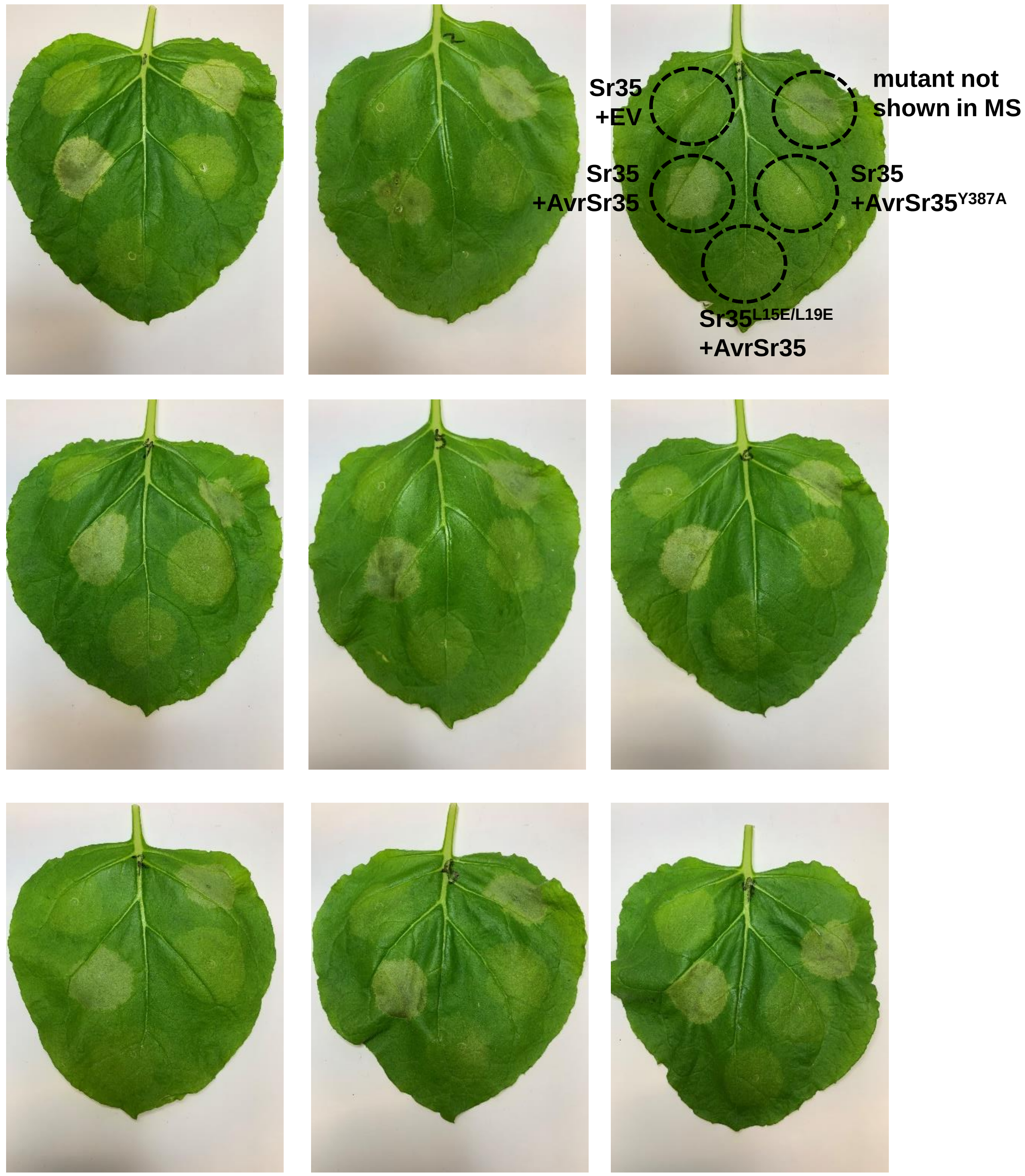

Supplemental Information 5 Tobacco cell death data of AvrSr35 mutants corresponding to Figure 4f

*Hv*Sh1 and *Ta*Sh1 chimeras

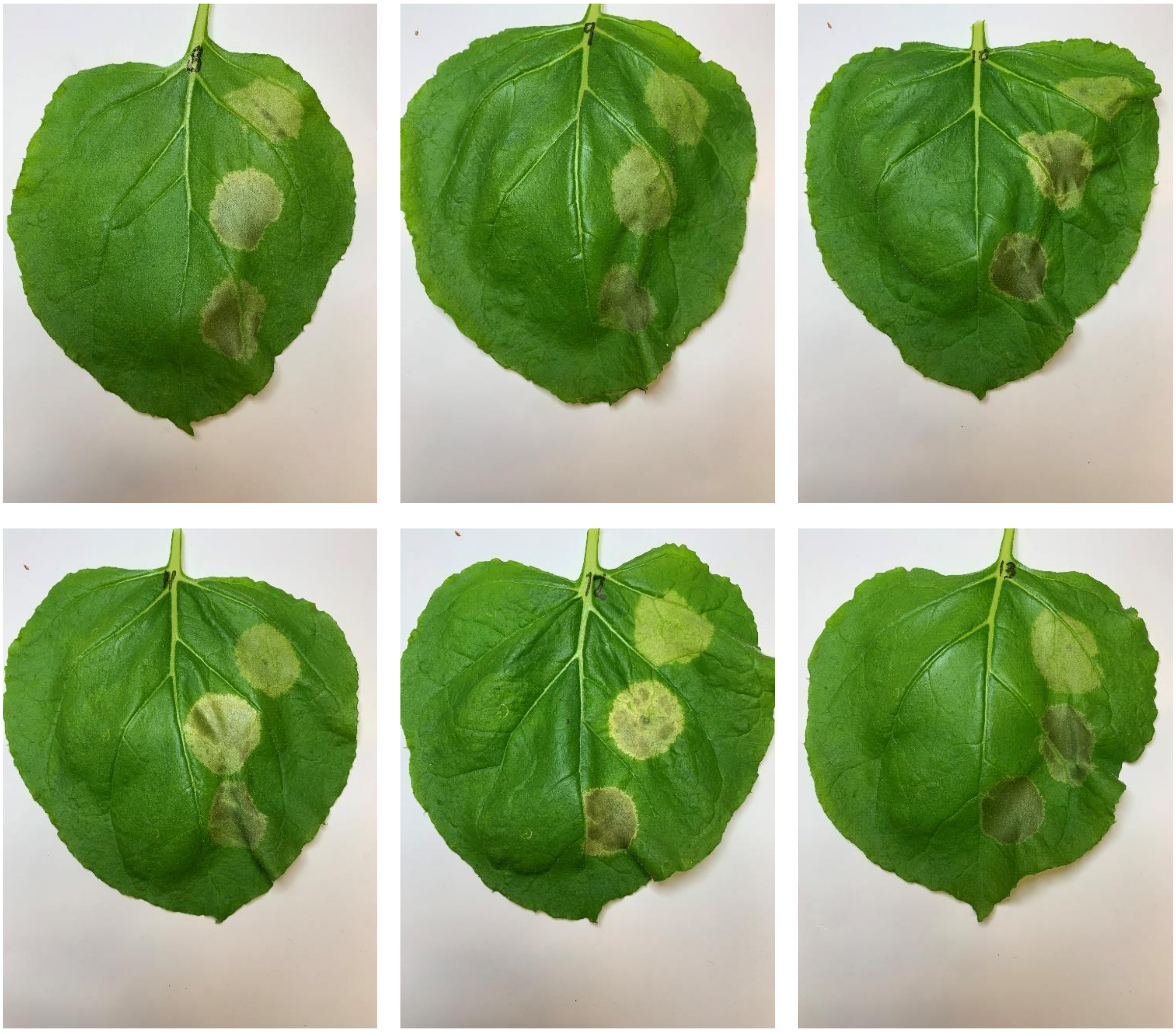

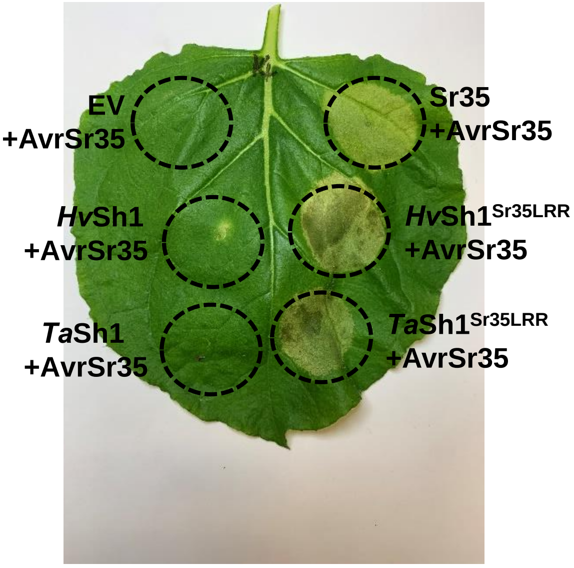

Supplemental Information 6 Tobacco cell death data of *Hv*Sh1 and *Ta*Sh1 chimeras corresponding to Figure 5c

*Hv*Sh1 and *Ta*Sh1 gain-of-function

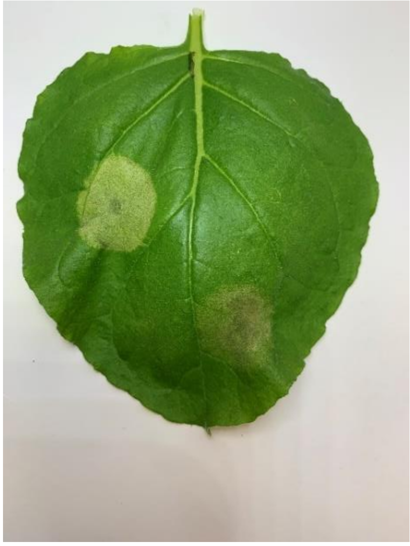

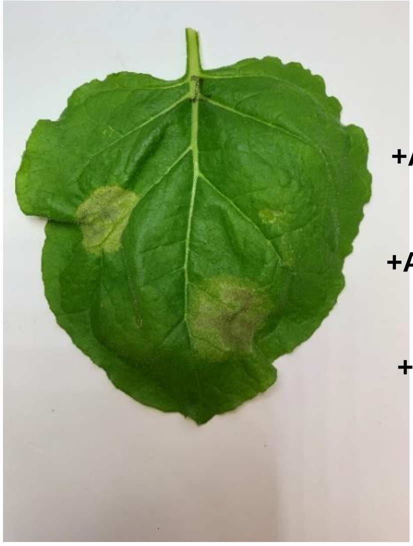

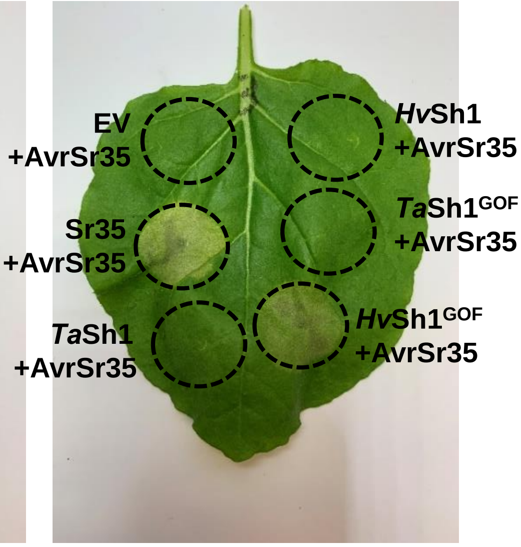

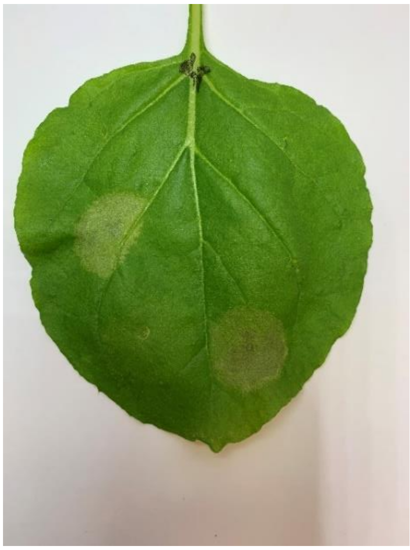

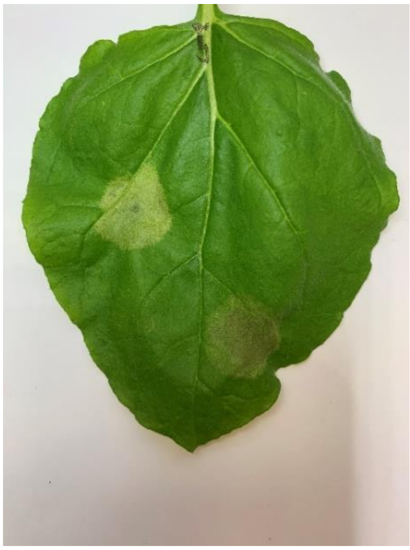

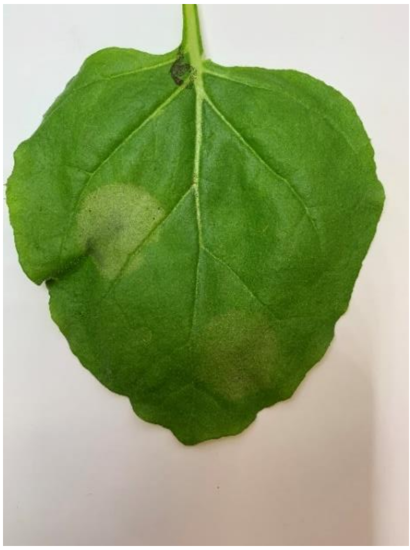

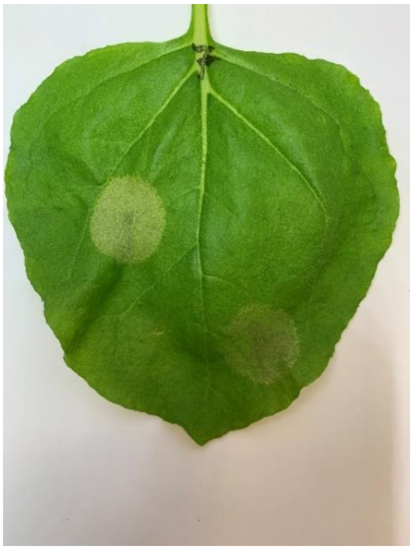

Supplemental Information 7 Tobacco cell death data of *Hv*Sh1 and *Ta*Sh1 gain-of-function corresponding to Figure 5g

*Hv*MLA chimeras

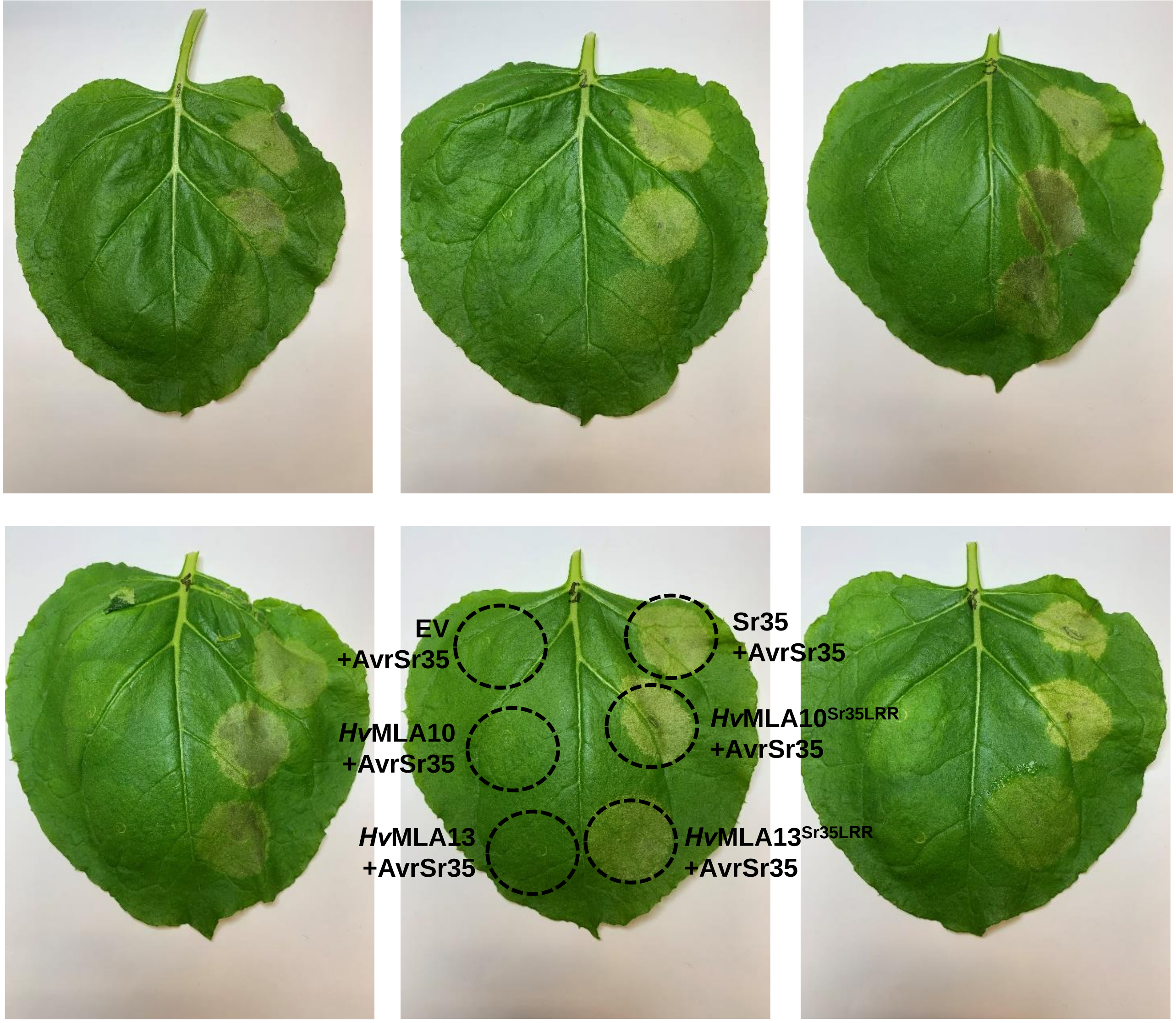

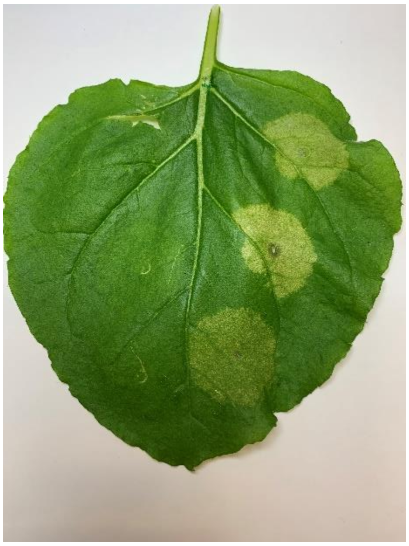

Supplemental Information 8 Tobacco cell death data of *Hv*MLA chimeras corresponding to Figure 5k

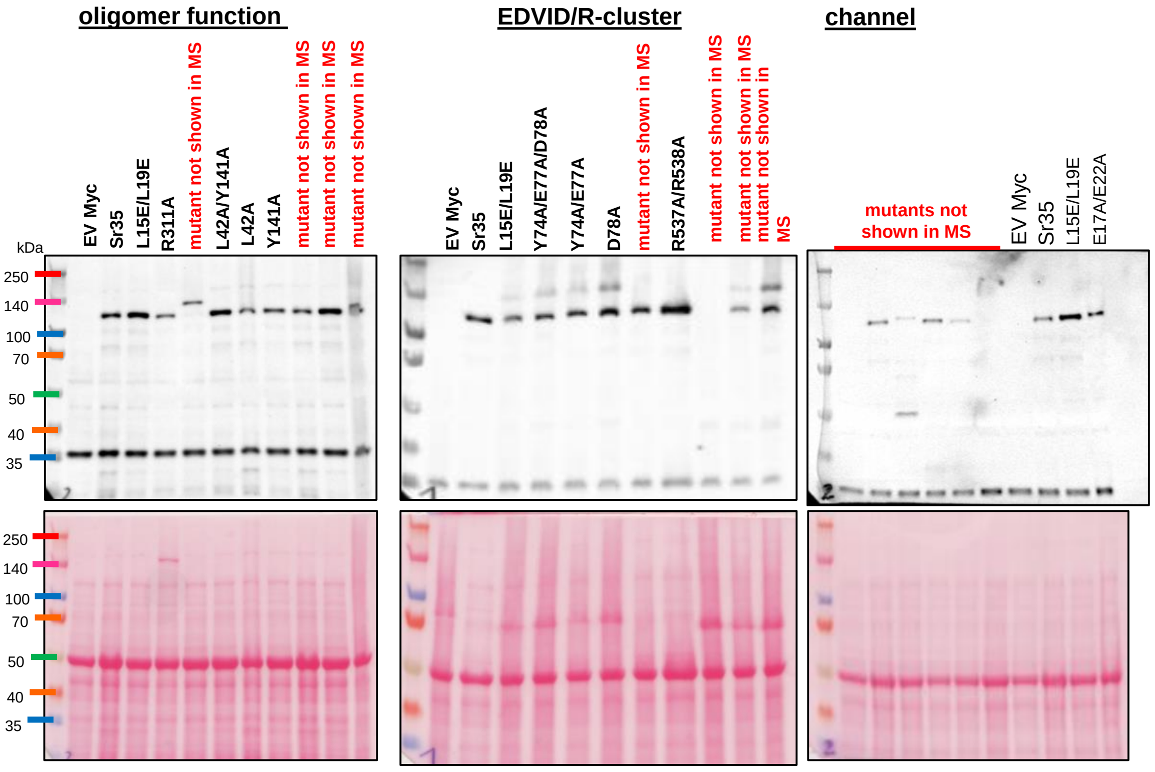

Supplemental Information 9 Uncropped western blots corresponding to Figure 2h, j (oligomer function and EDVID/R-cluster) and Figure 3e (channel).

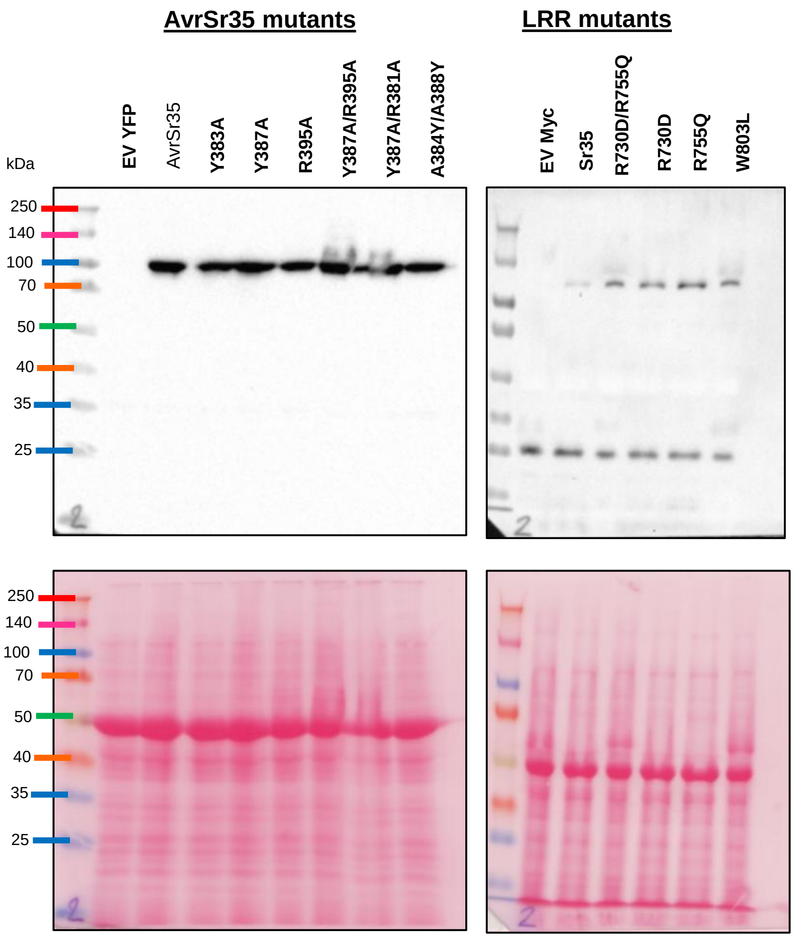

Supplemental Information 10 Uncropped western blots corresponding to Figure 3d, f

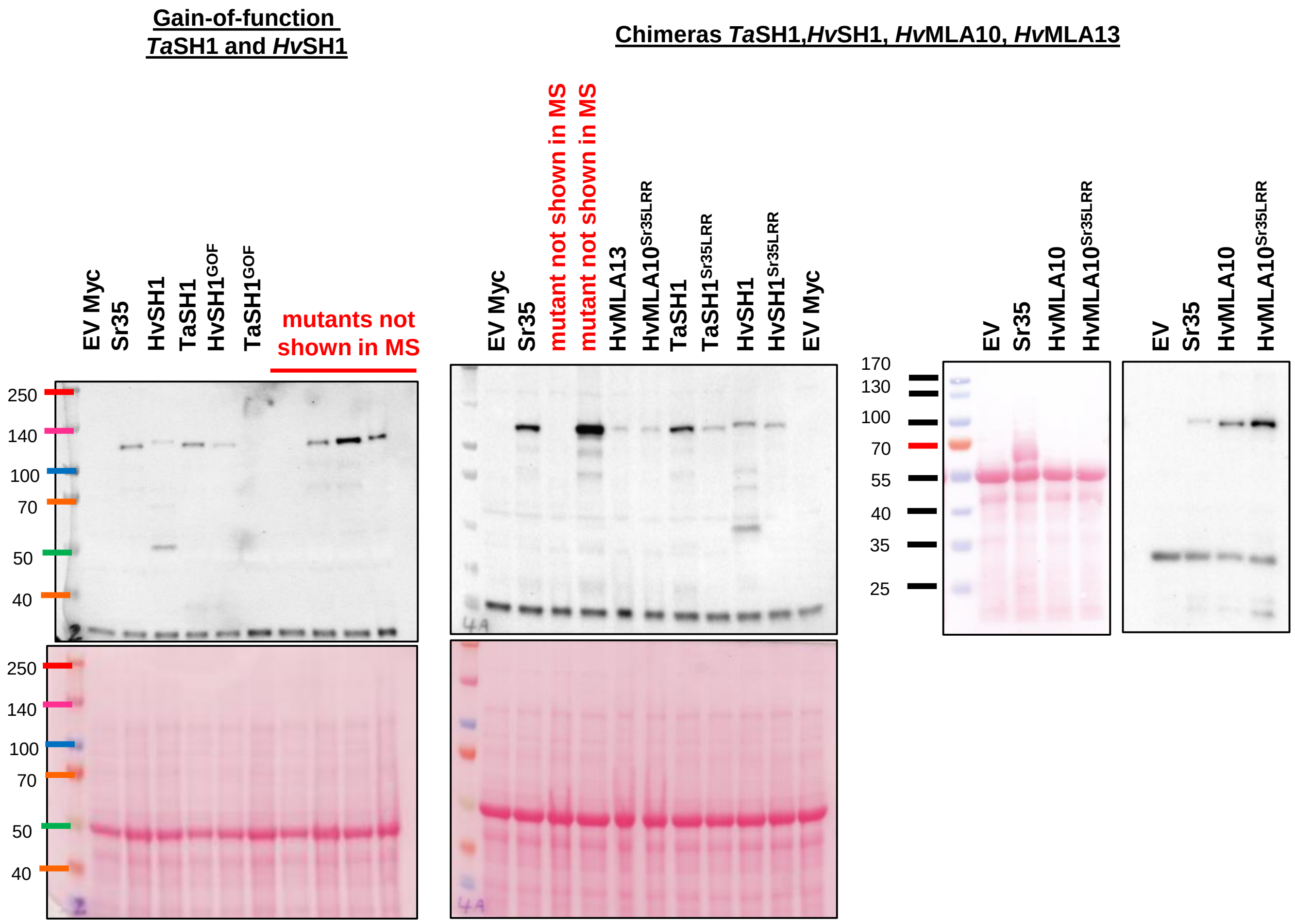

Supplemental Information 11 Uncropped western blots of Figure 5d, h, l
