# Supplementary material for: A wheat resistosome defines common principles of immune receptor channels
Source: Nature. 2022 Sep 26;610(7932):532–9. doi: 10.1038/s41586-022-05231-w (PMC9581773; doi:10.1038/s41586-022-05231-w)
Supplement: Supplementary file 2 — Reporting Summary [file 41586_2022_5231_MOESM2_ESM.pdf]

nature portfolio

Corresponding author(s): Jijie Chai, Alexander Förderer, Yuhang Chen

Last updated by author(s): Jun 25, 2022

Reporting Summary

Nature Portfolio wishes to improve the reproducibility of the work that we publish. This form provides structure for consistency and transparency in reporting. For further information on Nature Portfolio policies, see our Editorial Policies and the Editorial Policy Checklist.

Statistics

For all statistical analyses, confirm that the following items are present in the figure legend, table legend, main text, or Methods section.

n/a	Confirmed	
☐	☒	The exact sample size (*n*) for each experimental group/condition, given as a discrete number and unit of measurement
☐	☒	A statement on whether measurements were taken from distinct samples or whether the same sample was measured repeatedly
☐	☒	The statistical test(s) used AND whether they are one- or two-sided<br>*Only common tests should be described solely by name; describe more complex techniques in the Methods section.*
☐	☒	A description of all covariates tested
☐	☒	A description of any assumptions or corrections, such as tests of normality and adjustment for multiple comparisons
☐	☒	A full description of the statistical parameters including central tendency (e.g. means) or other basic estimates (e.g. regression coefficient) AND variation (e.g. standard deviation) or associated estimates of uncertainty (e.g. confidence intervals)
☐	☒	For null hypothesis testing, the test statistic (e.g. *F*, *t*, *r*) with confidence intervals, effect sizes, degrees of freedom and *P* value noted<br>*Give P values as exact values whenever suitable.*
☒	☐	For Bayesian analysis, information on the choice of priors and Markov chain Monte Carlo settings
☒	☐	For hierarchical and complex designs, identification of the appropriate level for tests and full reporting of outcomes
☒	☐	Estimates of effect sizes (e.g. Cohen's *d*, Pearson's *r*), indicating how they were calculated

Our web collection on statistics for biologists contains articles on many of the points above.

Software and code

Policy information about availability of computer code

Data collection	OC-725C oocyte clamp amplifier (Warner Instruments)<br>Titan Krios (Thermo Fisher Scientific), K3 Summit camera (Gatan)<br>luminometer (Centro, LB960)
Data analysis	EPU 2 (Thermo Fisher Scientific) 2.8.1.10REL<br>Relion 3.1<br>Coot 0.9<br>PHENIX 1.18.2<br>USCF Chimera 1.15<br>ChimeraX 1.15<br>OriginPro 2022<br>pClamp 10.6<br>Pymol Molecular Graphics System 1.7.2.1.<br>GraphPad Prism 8<br>Microsoft Office Software package (Excel) 2016<br>RStudio 2021.09.0 Build 351

For manuscripts utilizing custom algorithms or software that are central to the research but not yet described in published literature, software must be made available to editors and reviewers. We strongly encourage code deposition in a community repository (e.g. GitHub). See the Nature Portfolio guidelines for submitting code & software for further information.

Data

Policy information about availability of data

All manuscripts must include a data availability statement. This statement should provide the following information, where applicable:

- Accession codes, unique identifiers, or web links for publicly available datasets
- A description of any restrictions on data availability
- For clinical datasets or third party data, please ensure that the statement adheres to our policy

The atomic coordinates of the Sr35 resistosome have been deposited in the Protein Data Bank with the accession code 7XC2. The EM map for the local mask of Sr35 LRR in complex with AvrSr35 has been deposited in the EMDB with the accession code EMD-33111.
Sequences of TaSh1 and HvSh1 are available at NCBI under accession codes XP_044359492.1 (https://www.ncbi.nlm.nih.gov/protein/XP_044359492.1/) and KAE8803279.1 (https://www.ncbi.nlm.nih.gov/protein/KAE8803279.1), respectively.
Source data of tobacco infiltrations, western blots, insect cell viability and wheat protoplast cell death are provided with this manuscript.

Field-specific reporting

Please select the one below that is the best fit for your research. If you are not sure, read the appropriate sections before making your selection.

☒ Life sciences ☐ Behavioural & social sciences ☐ Ecological, evolutionary & environmental sciences

For a reference copy of the document with all sections, see nature.com/documents/nr-reporting-summary-flat.pdf

Life sciences study design

All studies must disclose on these points even when the disclosure is negative.

Sample size	No statistical methods were used to determine sample size. Sample size was chosen in accordance with the generally accepted standard of the respective scientific field.<br>The wheat protoplast experiments consisted of a total of five to six replicates. A maximum of two replicates were conducted per batch of wheat seedlings to encompass any variation due to the plant material. Five to six replicates of the wheat protoplast experiments were deemed sufficient due to the consistency of the results. The N. benthamiana infiltration experiments consisted of a minimum of seven biological replicates. Seven replicates are deemed sufficient due to any variation of protein expression in the leaves.<br>Sample size of Xenopus experiments was chosen based on previous literature (Bi et al. 2021) and deemed sufficient due to the low variation between technical and biological replicates.<br>More information is given in Statistics and reproducibility.
Data exclusions	No data were excluded from the analyses.
Replication	Generally, repetition was a measure taken to combat experimental variation. The plant data is deemed reproducible due to the use of different batches of plant material in both the wheat protoplast experiments and the N. benthamiana experiments. In addition, different Agrobacterium cultures were used to verify reproducibility of the N. benthamiana experiments.<br>The Xenopus data is deemed reproducible due to the use of different oocyte batches.<br>Expression from Sf21 insect cells was generally reproducible whenever the cell culture was in good health. Recovery of star-shaped particles varied according to culture health, baculovirus quality and experimenter performance in protein purification. Generally >20% star-shaped particles were recovered from Sr35 L15E/L19E and AvrSr35 co-expression.<br>Negative staining and cryo grid preparation are tricky procedures. Variablity of cryo grid preparation was reduced by using Vitrobot automation. Nevertheless, recovery of high quality electron microscopy samples varied significantly.
Randomization	Plant material was selected randomly from a given batch and analyzed equally. Randomization was deemed unnecessary as no sub-sampling was done.
Blinding	Blinding was not deemed relevant for our experiments given the nature of the reagents (plasmids, cRNA) or due to the protein purification work-flow (generally one large-scale purification at a time).

Reporting for specific materials, systems and methods

We require information from authors about some types of materials, experimental systems and methods used in many studies. Here, indicate whether each material, system or method listed is relevant to your study. If you are not sure if a list item applies to your research, read the appropriate section before selecting a response.

Materials & experimental systems

n/a	Involved in the study
	☒ Antibodies
	☒ Eukaryotic cell lines
☒	Palaeontology and archaeology
	☒ Animals and other organisms
☒	Human research participants
☒	Clinical data
☒	Dual use research of concern

Methods

n/a	Involved in the study
☒	ChIP-seq
☒	Flow cytometry
☒	MRI-based neuroimaging

Antibodies

Antibodies used	monoclonal mouse Anti-myc (R950-25, Invitrogen/Thermofisher)<br>c-Myc synthetic peptide: Glu-Gln-Lys-Leu-Ile-Ser-Glu-Glu-Asp-Leu-<br>Species reactivity: Tag. no further validation data available from manufacturer<br><br>polyclonal goat anti-mouse IgG-HRP (ab6728, Abcam), no further validation data available from manufacturer<br><br>polyclonal rabbit Anti-GFP; (pabg1, Chromtek), no further validation data available from manufacturer<br><br>polyclonal swine anti-rabbit IgG-HRP (PO399, Agilent DAKO)
Validation	Antibodies were not validated in-house and not validated by the manufacturer (see above).<br><br>However, antibodies were deemed reliable due to entries in 'antibodyregistry' under entries: monoclonal mouse Anti-myc (RRID = AB_2556560), polyclonal goat anti-mouse IgG-HRP (RRID = AB_955440) and polyclonal rabbit Anti-GFP (RRID = AB_2749857).<br><br>In addition, antibodies were validated by the use of an empty vector negative control (to control unspecific binding of antibodies to tobacco/agrobacterium proteins) and wild-type Sr35 (myc-tagged) or wild-type AvrSr35 (YFP-tagged) as positive controls on each western blot.

Eukaryotic cell lines

Policy information about cell lines

Cell line source(s)	Sf21 insect cell line (Invitrogen)
Authentication	none of the cell lines were authenticated
Mycoplasma contamination	cell line was not tested for mycoplasma contamination
Commonly misidentified lines<br>(See ICLAC register)	no commonly misidentified cell lines were used in this study

Animals and other organisms

Policy information about studies involving animals; ARRIVE guidelines recommended for reporting animal research

Laboratory animals	Xenopus laevis, female, age 2-2.5 years
Wild animals	no wild animals were used in this study
Field-collected samples	no field collected samples were used in this study
Ethics oversight	The animal study (Xenopus laevis) was reviewed and approved by Laboratory Animal Ethics Committee at Institute of Genetics and Developmental Biology, Chinese Academy of Sciences, Beijing, China with the approval ID AP2020029.

Note that full information on the approval of the study protocol must also be provided in the manuscript.
